# Bilateral chylothorax, chylopericardium and chylous ascitis

**DOI:** 10.4103/0970-2113.80330

**Published:** 2011

**Authors:** Anil Kashyap, Vineet Mahajan, Jagdeep Whig, Sushil Gupta

**Affiliations:** *Department of Pulmonary and Critical Care, Dayanand Medical College and Hospital, Ludhiana, Punjab, India*; 1 *Department of Critical Care, Dayanand Medical College and Hospital, Ludhiana, Punjab, India*

**Keywords:** Chylopericardium, chylothorax, Non-Hodgkin’s lymphoma

## Abstract

Non-Hodgkin’s lymphoma (NHL) can commonly present as chylothorax and rarely as chylopericardium. Here we are presenting a case of a 21-years-old female with bilateral chylothorax, chylopericardium and chylous ascites all together finally diagnosed to have NHL as the etiology. To the best of our knowledge, it has been reported very infrequently.

## INTRODUCTION

Chylothorax is an uncommon condition characterized by accumulation of fluid containing chylomicrons within the pleural cavity. Simultaneous accumulation of chyle in all serous cavities, though rare, can be associated with nontraumatic etiologies. By far, malignancy (especially lymphoma) represents the most frequent underlying cause. Management is mainly conservative with dietary modifications along with the treatment of underlying etiology.

## CASE REPORT

A 21-year-old female was admitted in the department of pulmonary medicine of Dayanand Medical College and Hospital with the complaints of dry cough, progressive breathlessness and orthopnea for last 20 days. She was also having throbbing headache along with slowly increasing facial and neck swelling for last 10 days.

General physical examination showed a pale female with gross facial and neck swelling associated with dilated superficial veins on neck and chest. There was no pulsus paradoxus and Kussmaul’s sign was absent. She was also having bilateral axillary lymphadenopathy (1 × 1 cm firm, mobile, non-tender and not fixed to underlying structures). Respiratory examination revealed grossly decreased movements bilaterally with stony dull percussion note. Breath sounds were absent in bilateral basal regions. On cardiovascular examination, S_1_ and S_2_ were diminished without any murmur. Per abdomen, shifting dullness was present without any organomegaly.

**Figure 1:**
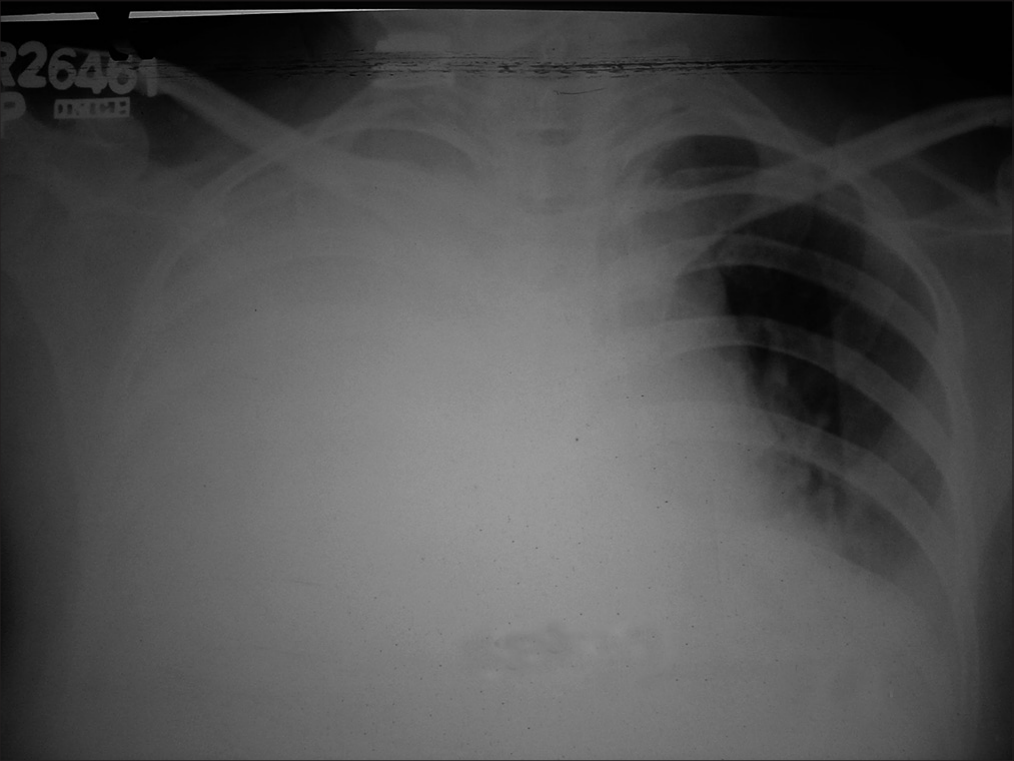
Chest radiograph showing bilateral pleural effusion and cardiomegaly

Routine blood investigations and coagulation profile were within normal limits. Chest radiograph was suggestive of bilateral pleural effusion and cardiomegaly [Figure 1]. As the patient was very dyspneic, pleural aspiration was done from both sides (about 0.5 l from right and 0.4 l from left milky fluid). Then we thought about the chylothorax. Clearing of pleural fluid by adding ethyl-ether into it leads to the exclusion of pseudochylothorax. Pleural fluid of both sides was sent for examination that revealed total leukocyte count: 600 cells/mm^3^, differential leukocyte count: neutrophils 85%, lymphocytes 15%, protein: 4.7 g%, sugar: 139 mg%, pleural fluid triglyceride: 725 mg %, pleural fluid cholesterol: 57 mg % on right side. Pleural fluid of left side revealed total leukocyte count 5800 cells/mm^3^, differential leukocyte count: neutrophils 60%, lymphocytes 40%, protein 4.8 g%, sugar 50 mg%, pleural fluid triglyceride: 426 mg%, pleural fluid cholesterol: 44.8 mg%. Fluid from both sides did not reveal any abnormal cell. Serum triglyceride and serum cholesterol were 129 mg% and 99 mg% respectively. The level of adenosine deaminase in pleural fluid was 32 IU/L. Pleural fluid culture was sterile in nature for pyogenic organisms. Bactec culture also did not reveal any growth of *Mycobacterium tuberculosis*.

Echocardiography was done which revealed moderate amount of pericardial effusion without any significant cardiac tamponade. Pericardial aspiration was done which was milky and exudative with high cholesterols. Biochemistry was in favor of chylopericardium. Abdominal ultrasound was suggestive of minimal ascites without any lymphadenopathy. In the light of confirmed bilateral chylothorax and chylopericardium, ascites was also thought to be chylous in origin. So, a diagnosis of superior vena cava syndrome, bilateral chylothorax, chylopericardium and chylous ascites was established but etiology remained unknown. She was put on conservative management along with low lipid diet but effusion re-accumulated and repeat thoracentesis was performed.

**Figure 2:**
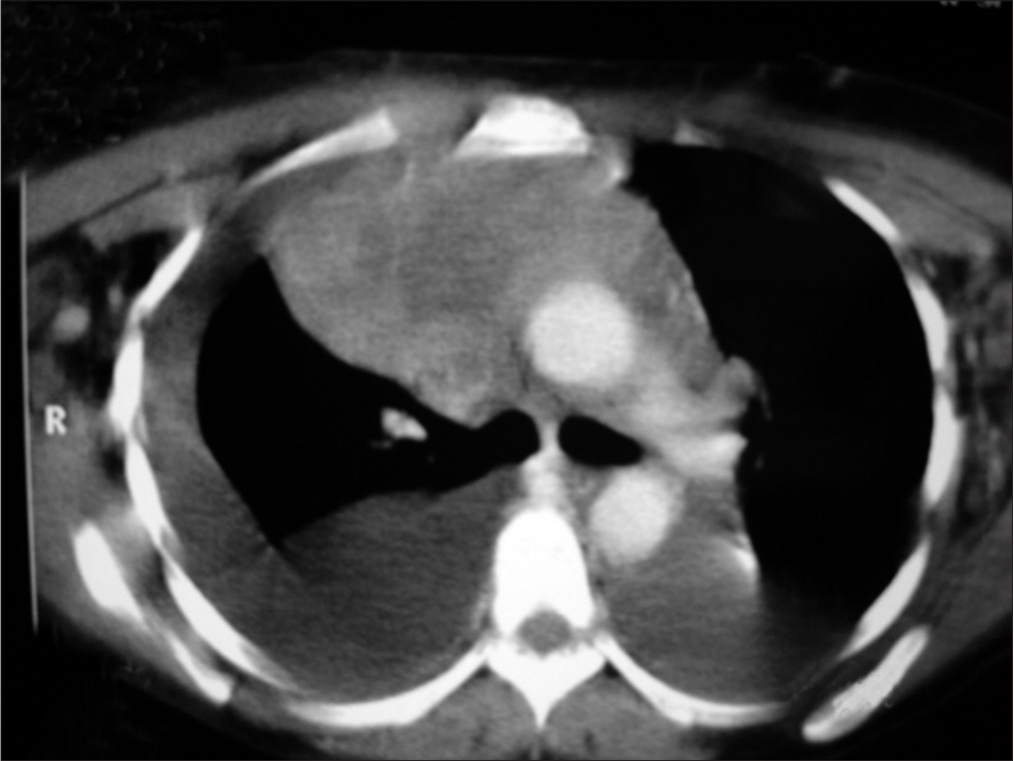
CT of thorax showing bilateral pleural effusion with large anterior mediastinal mass and superior vena cava thrombosis

**Figure 3:**
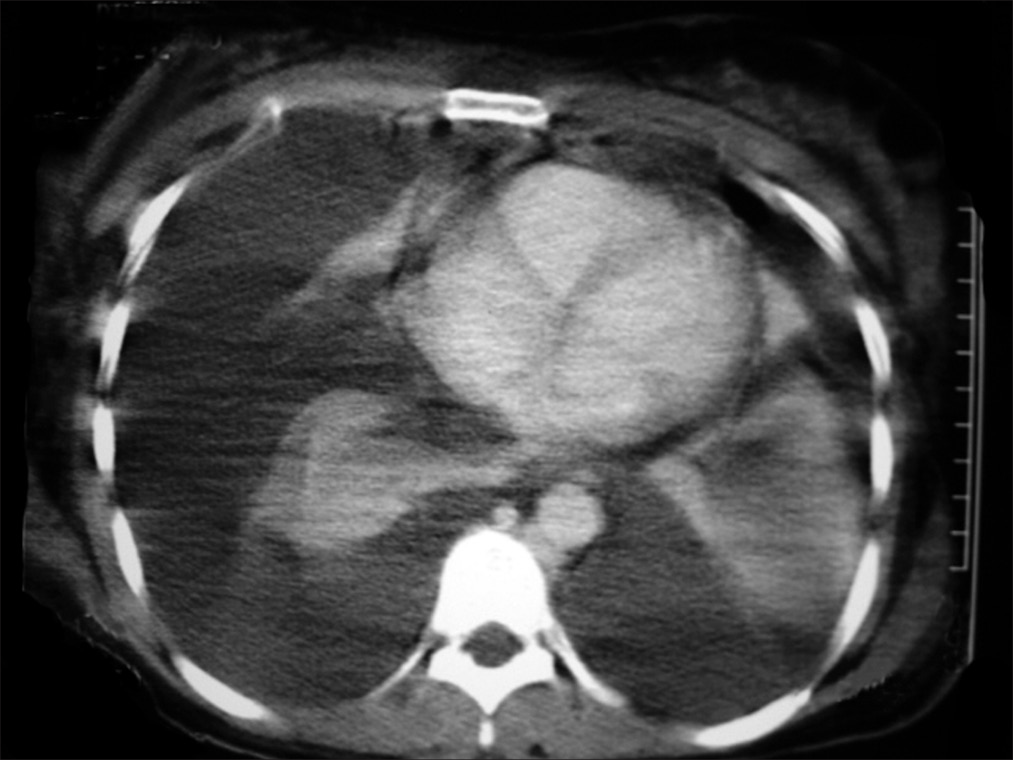
CT of thorax showing bilateral pleural and pericardial effusion

**Figure 4:**
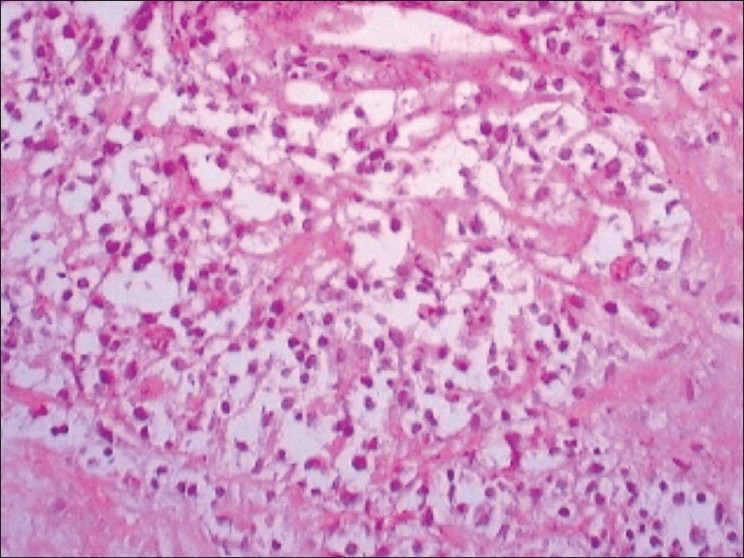
Trucut biopsy from the mass showing large round tumor cells with extensive ischemic necrosis suggestive of non-Hodgkin’s lymphoma (diffuse large B cell type)

Bone marrow aspiration was suggestive of normocellular, normocytic marrow. Fine needle aspiration cytology of left axillary lymph node was inconclusive. On symptomatic improvement, computed tomography of thorax was performed showing bilateral pleural effusion with large anterior mediastinal mass and superior vena cava thrombosis [Figure 2] and bilateral pleural with pericardial effusion [Figure 3]. Trucut biopsy from the mass was showing large round tumor cells with extensive ischemic necrosis suggestive of non-Hodgkin’s lymphoma (diffuse large B cell type) [Figure 4]. On flow cytometry, it was CD20 +ve and CD11 −ve, CD3 −ve. Considering the lymphoma as the etiology, R - CHOP (rituximab, cyclophosphamide, doxorubicin, onchovin and prednisolone) regimen was given. There was significant symptomatic and radiological improvement after first cycle of chemotherapy and patient is still in our follow up.

## DISCUSSION

Chylothorax and chylopericardium are characterized by fluid with a milky appearance due to high triglyceride content and the presence of chylomicrons as detected by lipoprotein electrophoresis. The etiology of chylothorax has been divided by Light[1] into four major categories: tumor, trauma, idiopathic, and miscellaneous. Lymphoma was the most common malignancy, occurring in three fourths of this group, and bronchogenic carcinoma was the next most common.[2] Most of the cases of chylothorax are unilateral, but bilateral chylothorax has also been reported in few cases.[3] Chylothorax is formed when the flow of chyle through the thoracic duct is disrupted causing elevated back pressure.[4]

Diagnosis of chylothorax is established by direct analysis of the pleural fluid. The fluid is characteristically “milky” in appearance, although not all milky effusions are chylous in nature and not all chylous effusions are milky. The triglyceride concentration greater than 110 mg/dl (in our case it was 725 mg%), a ratio of pleural fluid to serum triglyceride of greater than 1.0 (in our case it was 5.6), and ratio of pleural fluid to serum cholesterol of less than 1.0 (in our case it was 0.58) usually confirms chylothorax. Chylothorax will be excluded if pleural fluid triglyceride concentration is less than 50 mg/dl. However, in case of levels in between 50 and 110 mg/dl, a lipoprotein analysis of pleural fluid should be performed and demonstration of chylomicrons in fluid confirms the diagnosis of chylothorax.[5]

Chylous ascites is very rare and it occurs approximately in 1 in 20000 cases.[6,7] Chylopericardium can occur as isolated primary chylopericardium[8,9] or secondarily as a complication of cardiac surgery.[10] Benign superior vena cava occlusion can also present as bilateral chylothorax and chylopericardium which can be treated successfully with endovascular stent placement. Bilateral chylothorax and chylopericardium can also occur as a rare and severe complication of central venous catheterization which can be iatrogenic after placement of hemodialysis catheter[11] or in intravenous drug abusers[12] leading to extensive thrombosis within the venous system of the lungs.

The first step in the management of chylothorax demands a review of the history and physical examination.[13] Since lymphoma is the most common cause of chylothorax in the non-traumatic etiology, a computed tomography of the chest and abdomen should be performed to evaluate the mediastinum and abdominal lymphadenopathy. Mediastinal growth was obvious on computed tomography of thorax in our patient. Flow cytometric immunophenotyping has become a widely used laboratory procedure for diagnosis and sub-typing of lymphoma. It allows a more precise definition of individual cell types since the cells of interest are identified by a combination of physical characteristics and the use of multiple antibodies directly conjugated with different fluorochromes. Primary treatment in case of chylothorax should be directed toward correction of malnutrition and compromised immunological status which is due to the repeated pleural fluid aspiration of chyle with its high levels of protein, fat, electrolytes and lymphocytes.[14] The defect in thoracic duct often closes spontaneously in case of traumatic injury. In case of severe dyspnoea, placement of pleuro-peritoneal shunt or chest tube drainage is mandatory. If chylothorax persists for more than four weeks, consideration should be given to surgical exploration with ligation of the thoracic duct.[15] Other modalities including pleurodesis and pleurectomy may also be used. Finally, other nontraumatic chylous effusions may be amenable to medical therapy.[16]

In conclusion, early recognition of chylous leakage, prompt treatment with fat free and a medium chain triglycerides (MCT) - rich diet and good compliance are still found to be effective in the initial management of chylothorax and chylopericardium leading to favorable outcome without complications. Also, one should look for other potential sites of chylous origin apart from pleural cavity as appropriate and timely management is sometimes life saving.
